# Supplementary material for: Complexome profiling of the Chlamydomonas psb28 mutant reveals TEF5 as an early PSII assembly factor
Source: Plant Cell. 2025 Mar 17;37(6):koaf055. doi: 10.1093/plcell/koaf055 (PMC12164590; doi:10.1093/plcell/koaf055)
Supplement: koaf055_Supplementary_Data [file koaf055_supplementary_data.zip › psb28_interactive.zip!/plots/intensity/qualityPlots/peptides/SQD2_Cre01.g038550.t1.1.html]

### Cre01.g038550.t1.1

Trivial name: SQD2  
SeqCoverage [median]: 16.50  
Mass: 59.38 kDa  
  
 

| Sequence | MissedCleavage | Mass [Da] | wtIntensity | muIntensity | Score |
| --- | --- | --- | --- | --- | --- |
| EQPETFAGAR | 0 | 1104.52 | 0.00 | 1210.71 | 57.96 |
| GVDSEVFHPR | 0 | 1141.55 | 0.00 | 1260.32 | 53.33 |
| GYTLPGVDASSFR | 0 | 1368.67 | 0.00 | 901.20 | 59.43 |
| LTGGQPDRPTLVYVGR | 1 | 1727.93 | 563.07 | 7737.28 | 55.66 |
| NPGVGLAFVGDGPAR | 0 | 1425.74 | 0.00 | 2420.46 | 137.16 |
| QELQAAFK | 0 | 933.49 | 0.00 | 486.44 | 79.12 |
| VAQDALAAQPPQTGGLTPR | 0 | 1890.00 | 0.00 | 1019.90 | 68.24 |
|  |  |  | 563.1 | 15036.3 |  |
